# Soluble CD163 and TWEAK in early pregnancy gestational diabetes and later glucose intolerance

**DOI:** 10.1371/journal.pone.0216728

**Published:** 2019-05-09

**Authors:** Jonatan Dereke, Jacob Nilsson, Charlotta Nilsson, Helena Strevens, Mona Landin-Olsson, Magnus Hillman

**Affiliations:** 1 Lund University, Faculty of Medicine, Department of Clinical Sciences Lund, Diabetes Research Laboratory, Lund, Sweden; 2 Department of Paediatrics, Helsingborg Hospital, Helsingborg, Sweden; 3 Department of Obstetrics, Skåne University Hospital Lund, Lund, Sweden; 4 Department of Endocrinology, Skåne University Hospital Lund, Lund, Sweden; Stony Brook University Health Sciences Center School of Medicine, UNITED STATES

## Abstract

Gestational diabetes mellitus (GDM) is today universally diagnosed during late pregnancy. Treating hyperglycaemia during pregnancy reduces the risk of complications, the effect of interventions is however limited due to the late diagnosis. It is thus important to identify biomarkers reaching a high precision for GDM development in early pregnancy. Here we aim to investigate soluble CD163 (sCD163) and soluble tumour necrosis factor-like weak inducer of apoptosis (sTWEAK) in early pregnancy GDM and their association to the development of later glucose intolerance. In this case-control study, women diagnosed with GDM in early pregnancy (n = 70) at Lund University Hospital, Lund, Sweden in 2011–2015 were age- and BMI matched to pregnant volunteers without diabetes (n = 70) recruited in early pregnancy from maternal health care centres in 2014–2015. Plasma levels of sCD163 and sTWEAK were analysed using commercial ELISA. Plasma levels of sCD163 did not differ between patients with and without GDM in early pregnancy (p = 0.86), plasma levels of sTWEAK however was decreased in women with GDM (0.71 [0.4–1.75] ng/ml) compared to controls (1.38 [0.63–4.86] ng/ml; p = 0.003). Women with sTWEAK levels in the lowest tertile had an increased risk of GDM in early pregnancy (p = 0.014). Neither sCD163 nor sTWEAK were associated with later glucose intolerance in women with GDM. This study reports decreased levels of sTWEAK in women with early pregnancy GDM, independent of age and BMI. Neither sCD163 nor sTWEAK were found to be associated to later glucose intolerance.

## Introduction

Gestational diabetes mellitus (GDM) affects approximately 2% of all pregnant women in southern Sweden [1]. Pregnancies complicated by GDM reveal an increased risk of hypertensive disorders and future manifest diabetes for the mother. GDM may also have adverse effects on foetal development, increasing the risk of macrosomia, perinatal complications and the risk for future obesity in the offspring [2].

Increased levels of free fatty acids and advanced glycation end products, which lead to the production of pro-inflammatory cytokines from adipose tissue and subsequent chronic inflammation, are typical for GDM [3]. Inflamed adipose tissue causes monocyte infiltration and activation of tissue resident macrophages. The endocytic scavenger receptor CD163 is expressed on macrophages active in adipose tissue [4]. A large prospective study showed that increased serum levels of soluble CD163 (sCD163) could predict future development of type 2 diabetes independent of age and body mass index (BMI) [5]. Studies on sCD163 in GDM are few and inconclusive with one study reporting increased levels in GDM [6], while another found no difference compared to in pregnant women with normal glucose tolerance (NGT) [7].

Soluble tumour necrosis factor-like weak inducer of apoptosis (sTWEAK) exert multiple different cellular activities on a wide array of cells, which are important in regulating the anti-inflammatory/inflammatory balance, and has also been suggested as a ligand for CD163 [8, 9]. Studies made on circulating sTWEAK in diabetes report significantly decreased serum levels in both type 1 diabetes and type 2 diabetes [10, 11]. One study reported lower levels of sTWEAK in women with GDM compared to women with NGT [7].

GDM is today universally diagnosed during late pregnancy. Treating hyperglycaemia during pregnancy is known to reduce the risk of complications [12], but the effect of intervention is often limited due to the late diagnosis. Finding early pregnancy biomarkers capable of identifying women at increased risk for GDM could motivate earlier and more efficient interventions, minimising the risk for adverse outcomes [13]. Identifying GDM patients at increased risk of later glucose intolerance development is of importance and could act as an incentive for necessary lifestyle changes and pharmacological interventions in these women [14].

The aims of this study was to investigate plasma levels of sCD163 and sTWEAK in pregnant women with and without GDM in early pregnancy and their association to later glucose intolerance.

## Participants and methods

### Participants

In the catchment area of Lund University Hospital, Lund, Sweden, pregnant women are offered an OGTT as part of a general GDM screening. The glucose load for the OGTT is 75-g following overnight fast and a 2-hour capillary plasma glucose value above 10mmol/l was used as the diagnostic cut-off for GDM at the time of patient recruitment as recommended by the European Association for the Study of Diabetes (EASD) [15]. Between 2011 and 2015 a total of 519 pregnant women were diagnosed with GDM with 140 receiving the diagnosis in early pregnancy. The patients included in this study were diagnosed in early pregnancy (gestational age 14 ± 4 weeks) (n = 70). Information regarding family history of diabetes, previous GDM and later development of glucose intolerance was retrieved when available from the patients’ primary and/or secondary records both by studying journals and ICD-10 codes in 2018. Thirteen (19%) of the patients developed glucose intolerance up to four years after delivery. The mean time to development was 1.7 ± 1.1 years. Age and BMI matched pregnant controls (n = 70) were recruited at their first visit to the maternal health care centres (around week 12) from the same geographical region in 2014–2015. Controls with previously diagnosed diabetes or GDM were not included in this study. Blood samples were drawn into ethylenediaminetetraacetic acid (EDTA) plasma tubes and centrifuged at 2000 x g for 10 minutes. The soluble component was separated from the blood cells and stored at -70°C until time of analysis.

This study was approved by the regional ethical committee in Lund (Regionala etikprövningsnämnden Lund) (849/2005, 2014/78 and 2014/744) and performed in accordance with the principles of the 1964 Declaration of Helsinki. All participants received oral and written information about this study before providing written informed consent.

### Biochemical analyses

Plasma levels of sCD163 and sTWEAK were measured using commercially available DuoSet enzyme linked immuno-sorbent assay (ELISA) kits (R&D Systems, Minneapolis, Minnesota, USA) and optimised for human plasma. The analyses were run according to the manufacturer’s instructions. Samples were diluted 1:200 and 1:5 respectively for the sCD163 and sTWEAK analysis. The intra-assay coefficient of variation was 8.7% for sCD163 and 7.8% for sTWEAK respectively. All samples were run as duplicates, and patient and control samples were alternated to minimise bias caused by intra-assay variation.

### Statistical analyses

Mean ± standard deviation (SD) were used to present data for continuous variables where normality was accepted and median followed by interquartile range in brackets where normality was rejected. Student’s t-test or the Mann-Whitney *U*-test were used to compare differences between groups. Spearman’s rho was used in order to assess the degree of correlation between variables. Odds-ratios (OR) were calculated for the association of GDM with regard to plasma sTWEAK tertiles. P-values less than 0.05 were considered statistically significant. All statistical analyses were performed using MedCalc Statistical Software version 18.9 (MedCalc Software bvba, Ostend, Belgium; http://www.medcalc.org; 2018).

The data set containing raw data of sCD163, sTWEAK and postpartum diabetes development is provided as Supporting Information (S1 Dataset).

## Results

No difference in plasma levels of sCD163 could be observed between women with or without GDM diagnosed in early pregnancy (318 [246–395] ng/ml and 321 [243–432] ng/ml respectively; p = 0.86). Plasma levels of sTWEAK were however decreased in women with early pregnancy GDM (0.71 [0.4–1.75] ng/ml) compared to controls (1.38 [0.63–4.86] ng/ml; p = 0.003). Plasma sTWEAK levels were not associated to participant age (p = 0.61) or BMI (p = 0.55), and no correlation between sCD163 and sTWEAK could be observed (p = 0.86). There was no association between sCD163 (p = 0.16) or sTWEAK (p = 0.27) and glucose intolerance development following GDM in this study. Women with GDM had more often a family history of diabetes (57% (36/63)) compared to controls (31% (22/70; p = 0.003). Neither sCD163 nor sTWEAK levels were found to differ in women with or without a family history of diabetes (p = 0.48 and p = 0.27 respectively). Some of the patients have had previous GDM (10% (6/59)). No association could be observed between previous GDM and sCD163 (p = 0.76) or sTWEAK (p = 0.74) levels.

In order to increase reproducibility through different modes of analysis, sTWEAK levels were arranged into tertiles (T_1_-T_3_). Plasma sTWEAK levels in the lowest tertile (T_1_ (<0.63ng/ml)) were associated with an increased risk of GDM (OR: 2.48, CI^95^ (1.20–5.10); p = 0.014), while sTWEAK levels in the highest tertile T_3_ (≥1.75ng/ml) showed a tendency for a decreased risk of GDM (OR: 0.49, CI^95^ (0.24–1.00; p = 0.05) (Table 1).

**Table 1:** Pregnant women in early pregnancy with plasma levels of sTWEAK in the lowest tertile had increased risk of being diagnosed with GDM. A tendency of decreased risk of GDM could be seen in the highest sTWEAK tertile.

sTWEAK tertile	Controls (n = 70)	GDM (n = 70)	OR (CI^95%^)	P-value
T_1_ (<0.63ng/ml)	17 (24.3%)	31 (44.3%)	2.48 (1.20–5.10)	0.014
T_2_ (0.63–1.71ng/ml)	24 (34.3%)	21 (30%)	0.82 (0.40–1.67)	0.59
T_3_ (≥1.75ng/ml)	29 (41.4%)	18 (25.7%)	0.49 (0.24–1.00)	0.05

## Discussion

This study has investigated sCD163 and sTWEAK levels in women diagnosed with GDM in early pregnancy. While no difference in sCD163 levels could be observed, decreased levels of sTWEAK were associated with the development of GDM. A previous study has found increased levels of sCD163 at delivery in women diagnosed with GDM in late pregnancy [6], while another study where sCD163 was measured in late pregnancy did not observe a difference when compared to women with NGT [7] which is in accordance with our findings in early pregnancy. The study by Bari et al. [6] investigated sCD163 levels at parturition in women with elective caesarean section in a small group of GDM patients (n = 18) and BMI matched controls (n = 20). They suggested sCD163 to be a significant predictor of GDM, although the precision was not presented. The late sampling and small sample size could be a reason for the discrepancy among the studies. Adipose tissue explants and placenta showed increased sCD163 release in GDM and the placenta was suggested to be a significant source of sCD163 in GDM. Simon-Muela et al. measured sCD163 levels in late pregnancy GDM (n = 66) and NGT (n = 71) [7]. They found sCD163 levels to be associated to a poor metabolic profile, but could not find any significant difference in sCD163 levels between the groups. In contrast to the study by Bari et al, sCD163 release from adipose tissue and placenta was not studied by Simon-Muela et al. nor by this study.

GDM is a condition characterised by chronic inflammation and decreased sTWEAK levels have been observed in diseases associated with chronic inflammation and insulin resistance [10, 11]. Circulating sTWEAK was in this study decreased in early pregnancy GDM independent of participant age and BMI and family history of diabetes. Our results are in agreement with a Spanish study made in later pregnancy [7]. Membrane bound CD163 has been reported to bind sTWEAK in circulation [8]. We could however, in contrast to the Spanish study [7], not observe any correlation between circulating sCD163 and sTWEAK. By arranging the sTWEAK levels into tertiles, we facilitate the possibility to reproduce the findings in this study. Women with sTWEAK levels in the lowest tertile had significantly increased risk of GDM, while there was a tendency for a decreased risk of GDM with sTWEAK levels in the highest tertile. In this study we also sought to determine any associations between sCD163, sTWEAK and the development of glucose intolerance following a pregnancy complicated by GDM. Neither sCD163 nor sTWEAK were however found to be associated to such development, possibly explained by the low number of patients who developed later glucose intolerance.

A strength with this study is that the GDM patients included are part of a unique screening program that enables all pregnant women in Lund to undergo an OGTT, resulting in a heterogeneous group of participants. The women included as controls are matched in age and BMI, and they are recruited from the same geographical region as the patients, further minimising confounding effects. Limitations include a limited number of participants and lacking the possibility for a follow-up during late pregnancy. A power calculation suggests 145 participants per group for sTWEAK and 759 for sCD163 with an alpha value of 0.05 and beta value of 0.8 for later glucose intolerance. It is important to notice however that the distribution for these molecules in non-parametric which may skew the power calculation.

## Conclusions

This study reports decreased levels of sTWEAK in women with early pregnancy GDM, independent of age and BMI. No difference could be observed with regard to sCD163 levels, and neither sTWEAK nor sCD163 was associated with later glucose intolerance. A future prospective cohort study should focus on elucidating the potential for sTWEAK to be used as an early pregnancy pre-screening biomarker for GDM development as this could possibly alleviate the need for onerous screening procedures in women with low risk for developing hyperglycaemia. More focus should also lie in earlier identification of women at risk of developing GDM as this could motivate more efficient treatment interventions at an earlier time point, minimizing adverse outcomes.

## Supporting information

S1 Dataset Minimal data set underlying the results of this study. (XLSX)

## References

[1] Ignell C, Claesson R, Anderberg E, Berntorp K. Trends in the prevalence of gestational diabetes mellitus in southern Sweden, 2003–2012. Acta Obstet Gynecol Scand. 2014;93(4):420–4. 10.1111/aogs.12340 24450766

[2] Buchanan TA, Xiang AH, Page KA. Gestational diabetes mellitus: risks and management during and after pregnancy. Nature reviews Endocrinology. 2012;8(11):639–49. 10.1038/nrendo.2012.96 22751341 PMC4404707

[3] Lappas M. Activation of inflammasomes in adipose tissue of women with gestational diabetes. Molecular and cellular endocrinology. 2014;382(1):74–83. 10.1016/j.mce.2013.09.011 24055273

[4] Zeyda M, Farmer D, Todoric J, Aszmann O, Speiser M, Gyori G, et al Human adipose tissue macrophages are of an anti-inflammatory phenotype but capable of excessive pro-inflammatory mediator production. Int J Obes (Lond). 2007;31(9):1420–8. 17593905 10.1038/sj.ijo.0803632

[5] Moller HJ, Frikke-Schmidt R, Moestrup SK, Nordestgaard BG, Tybjaerg-Hansen A. Serum Soluble CD163 Predicts Risk of Type 2 Diabetes in the General Population. Clinical Chemistry. 2011;57(2):291–7. 10.1373/clinchem.2010.154724 21106861

[6] Bari MF, Weickert MO, Sivakumar K, James SG, Snead DR, Tan BK, et al Elevated soluble CD163 in gestational diabetes mellitus: secretion from human placenta and adipose tissue. PLoS One. 2014;9(7):e101327 10.1371/journal.pone.0101327 24983948 PMC4077809

[7] Simon-Muela I, Llaurado G, Chacon MR, Olona M, Naf S, Maymo-Masip E, et al Reduced circulating levels of TWEAK are associated with Gestational Diabetes Mellitus. European journal of clinical investigation. 2015;45(1):27–35. 10.1111/eci.12375 25443800

[8] Bover LC, Cardo-Vila M, Kuniyasu A, Sun J, Rangel R, Takeya M, et al A previously unrecognized protein-protein interaction between TWEAK and CD163: potential biological implications. J Immunol. 2007;178(12):8183–94. 17548657 10.4049/jimmunol.178.12.8183

[9] Vendrell J, Chacón MR. TWEAK: A New Player in Obesity and Diabetes. Frontiers in immunology. 2013;4:488 10.3389/fimmu.2013.00488 24416031 PMC3874549

[10] Kralisch S, Ziegelmeier M, Bachmann A, Seeger J, Lossner U, Bluher M, et al Serum levels of the atherosclerosis biomarker sTWEAK are decreased in type 2 diabetes and end-stage renal disease. Atherosclerosis. 2008;199(2):440–4. 10.1016/j.atherosclerosis.2007.10.022 18054361

[11] Llaurado G, Gonzalez-Clemente JM, Maymo-Masip E, Subias D, Vendrell J, Chacon MR. Serum Levels of TWEAK and Scavenger Receptor CD163 in Type 1 Diabetes Mellitus: Relationship with Cardiovascular Risk Factors. A Case-Control Study. Plos One. 2012;7(8):e43919 10.1371/journal.pone.0043919 22937125 PMC3427173

[12] American Diabetes Association. 13. Management of Diabetes in Pregnancy. Diabetes Care. 2017;40(Supplement 1):S114–S9. 27979900 10.2337/dc17-S016

[13] Powe CE. Early Pregnancy Biochemical Predictors of Gestational Diabetes Mellitus. Curr Diab Rep. 2017;17(2):12 10.1007/s11892-017-0834-y 28229385

[14] Ratner RE, Christophi CA, Metzger BE, Dabelea D, Bennett PH, Pi-Sunyer X, et al Prevention of diabetes in women with a history of gestational diabetes: effects of metformin and lifestyle interventions. J Clin Endocrinol Metab. 2008;93(12):4774–9. 10.1210/jc.2008-0772 18826999 PMC2626441

[15] Lind T, Phillips PR. Influence of pregnancy on the 75-g OGTT. A prospective multicenter study. The Diabetic Pregnancy Study Group of the European Association for the Study of Diabetes. Diabetes. 1991;40 Suppl 2:8–13. 1748272 10.2337/diab.40.2.s8

